# Mechanistic Links Between Natural Bioactive Molecules and Tumor Immune Microenvironment States in PD-1/PD-L1 Resistance

**DOI:** 10.3390/biomedicines14091955

**Published:** 2026-08-30

**Authors:** Huiya Cheng, Meilun Chen, Min Xiao, Heteng Zhang, Zitong Zhang, Dongyue Jiang, Arabella H. Wan, Qiaoping Wang, Guohui Wan

**Affiliations:** 1 School of Pharmaceutical Sciences, Sun Yat-sen University, Guangzhou 510006, China; chenghy25@mail2.sysu.edu.cn (H.C.); chenmlun3@mail2.sysu.edu.cn (M.C.); xiaom9@mail2.sysu.edu.cn (M.X.); zhanght37@mail2.sysu.edu.cn (H.Z.); zhangzt53@mail2.sysu.edu.cn (Z.Z.); jiangdy8@mail2.sysu.edu.cn (D.J.); 2 Department of Pathology, The First Affiliated Hospital of Sun Yat-sen University, Guangzhou 510080, China; 3 The Affiliated Stomatological Hospital of Nanjing Medical University, Nanjing 210029, China

**Keywords:** PD-1/PD-L1 resistance, tumor immune microenvironment, natural bioactive molecules, immune checkpoint blockade, evidence assessment, translational barriers, biomarkers

## Abstract

Programmed cell death protein 1/programmed death ligand 1 (PD-1/PD-L1) blockade can produce durable responses, but primary and acquired resistance are common. Treatment outcome is influenced by antigen presentation, T-cell localization, suppressive myeloid populations, metabolic stress, and the gut microbiome, all of which shape the tumor immune microenvironment (TIME). This review examines natural bioactive molecules in relation to these resistance features rather than grouping them by chemical class. Castalagin/camu-camu, ginseng polysaccharides, and ginsenoside Rh2 have the clearest preclinical evidence from direct PD-1/PD-L1-combination studies; evidence for the curcumin-gasdermin E (GSDME) axis comes from one recent study. Demethylzeylasteral has a well-supported ubiquitin-specific peptidase 22 (USP22)-PD-L1 degradation mechanism, but the reported antibody combination used cytotoxic T-lymphocyte-associated protein 4 (CTLA-4) rather than PD-1/PD-L1. ACT001, berberine, baicalein, and several other candidates are supported mainly by indirect evidence of PD-L1 regulation or TIME remodeling. The translational value of these findings depends on exposure, target engagement, model selection, biomarker design, and material quality. Relating each candidate to a defined resistance setting helps distinguish promising combinations from mechanistic leads that still require direct testing.

## 1. Introduction

Blockade of the PD-1/PD-L1 axis is an established therapeutic strategy across multiple solid tumors. Clinically used PD-1 inhibitors include pembrolizumab, nivolumab, cemiplimab, and dostarlimab, whereas PD-L1 inhibitors include atezolizumab, durvalumab, and avelumab. PD-L1 immunohistochemistry informs treatment selection in selected non-small-cell lung cancer, head and neck squamous cell carcinoma, gastric or gastroesophageal junction adenocarcinoma, esophageal cancer, cervical cancer, urothelial carcinoma, triple-negative breast cancer, and epithelial ovarian, fallopian-tube, or primary peritoneal carcinoma settings. However, the assay, scoring system, cutoff, line of therapy, and combination regimen are indication specific. PD-L1 is therefore better regarded as a context-dependent predictive biomarker than as a universal prognostic marker, and PD-1 expression itself is not generally used as a tumor-cell threshold for treatment selection. Approval status and biomarker requirements also vary by jurisdiction and treatment setting; current regional product labels and companion-diagnostic documentation should therefore be consulted [1,2].

PD-1 blockade also has an important role in selected hematologic malignancies, particularly classical Hodgkin lymphoma and primary mediastinal large B-cell lymphoma. In these diseases, 9p24.1 alterations and increased programmed death ligand 1 (PD-L1) and programmed death ligand 2 (PD-L2) expression provide a distinct biological context [3,4]. Their tissue architecture, malignant-cell composition, and immune ecosystem differ from those of solid tumors. The present review therefore focuses primarily on solid tumors; hematologic malignancies are noted only to define the clinical scope and are not merged with the solid-tumor TIME framework.

Within solid tumors, treatment outcome is not determined solely by PD-L1 expression on cancer cells. It is also shaped by antigen presentation, the spatial distribution of T cells, stromal barriers, suppressive myeloid populations, immunometabolism, and the gut microbiome [5,6,7,8]. These components interact dynamically to determine whether checkpoint blockade can generate effective antitumor immunity. Transforming growth factor-β (TGF-β)-associated fibroblast signaling can retain CD8+ T cells in stromal regions and impair the efficacy of PD-L1 blockade, whereas β-catenin activation can reduce dendritic-cell recruitment and generate an immune-desert phenotype resistant to anti-PD-1 therapy [9,10]. C-X-C motif chemokine ligand 12 (CXCL12) derived from fibroblast activation protein-positive cancer-associated fibroblasts can restrict T-cell access to cancer cells, and the activity of combined C-X-C motif chemokine receptor 4 (CXCR4) inhibition and PD-L1 blockade indicates that spatial exclusion is itself an actionable resistance mechanism [11]. In parallel, monocarboxylate transporter 4 (MCT4)-dependent lactate export and an acidic metabolic milieu can suppress immune infiltration and T-cell function, placing metabolic restriction within the TIME-level biology of resistance [12].

Natural bioactive molecules are of interest because selected materials may modify the microbiome, inflammatory signaling, PD-L1 stability, T-cell recruitment, or immune metabolism; natural origin alone does not confer immunotherapeutic activity. They should be considered investigational adjuncts or mechanism probes, not substitutes for approved PD-1/PD-L1 antibodies. In a single-arm proof-of-concept study of patients with anti-PD-1-refractory melanoma, responder-derived fecal microbiota transplantation (FMT) plus pembrolizumab provided clinical benefit in 6 of 15 treated patients. The study was single-arm and did not establish a comparative benefit in overall or progression-free survival [13]. Preclinical natural-molecule combination studies are appraised in Section 3; they do not by themselves demonstrate clinical resistance reversal [14,15,16,17].

Many reviews of natural products and immune checkpoint inhibitor (ICI) therapy organize evidence by chemical class or compound. That format can obscure the translational questions: whether the tested material reaches the relevant tissue or immune niche, whether target engagement and causality have been shown, whether the study used an immunocompetent PD-1/PD-L1 combination model, and whether the proposed activity matches a defined TIME bottleneck. This review therefore separates direct PD-1/PD-L1 combination studies from mechanisms tested with another checkpoint, single-agent PD-L1 regulation, and delivery-only findings [18,19].

We use a narrative, mechanism-oriented approach to compare the evidence for each candidate with the solid-tumor TIME feature it is proposed to affect. The article is not a systematic review or meta-analysis. The analysis begins with five recurring resistance settings: failed priming or immune desert, immune exclusion, myeloid or metabolic restriction, microbiome-linked immune tone, and adaptive regulation of PD-L1 [5,6,7,8]. Senescence-associated secretory signaling is treated as a cross-state overlay rather than a coequal TIME subtype. Evidence for each resistance mechanism is considered separately from evidence that a natural molecule modifies it.

Studies are interpreted according to what they directly tested. We distinguish human studies, immunocompetent mouse models, other murine models, in vitro experiments, and delivery-only studies. Direct combination evidence is weighted according to monotherapy controls, immune readouts, causal perturbation, exposure, and target engagement. Single-agent tumor studies, immunodeficient xenografts, abstract-only reports, docking, and network pharmacology provide narrower evidence and are not treated as proof of checkpoint sensitization. In this review, sensitization requires a model with demonstrably poor baseline response or acquired resistance, prespecified monotherapy and combination contrasts, and evidence that the candidate improves checkpoint response through the proposed mechanism. A combination that outperforms both monotherapies is not described as pharmacologic synergy unless a formal interaction or additivity analysis supports that term.

**Figure 1:**
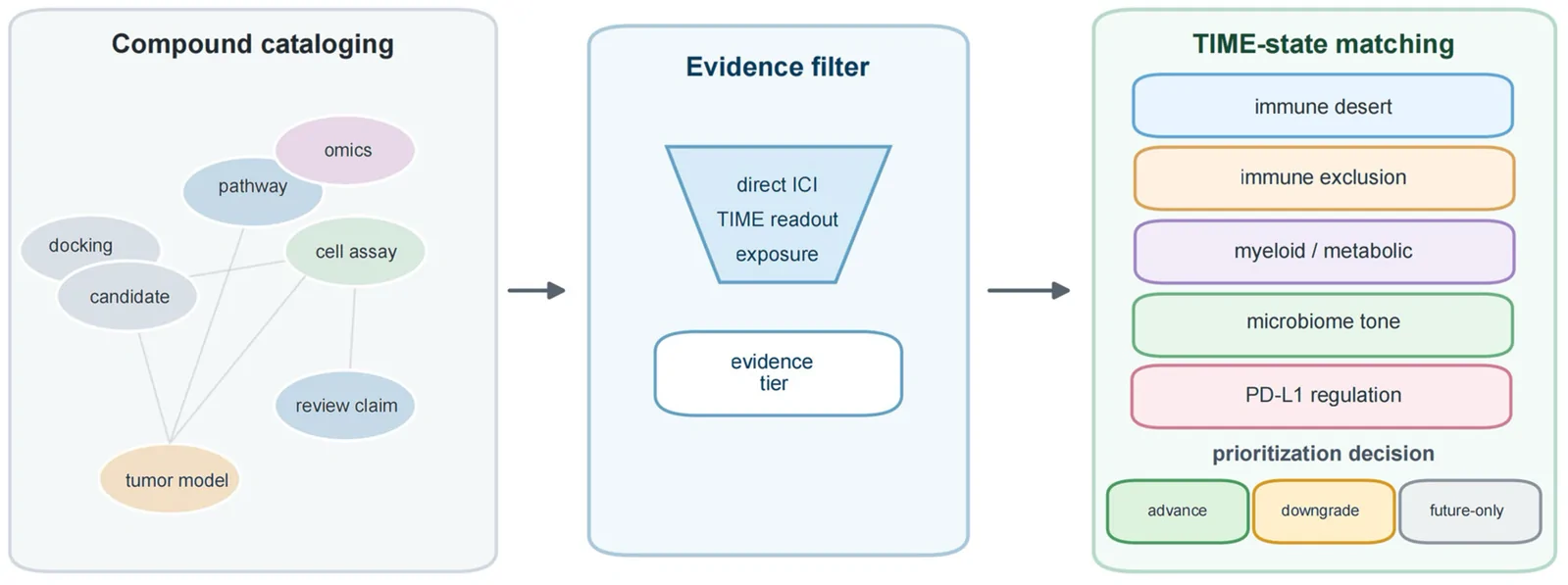
Organization of the review. Candidate molecules are evaluated according to evidence strength and their relationship to defined TIME features.

The discussion first defines the PD-1/PD-L1 inhibitory axis and TIME features associated with treatment failure. Natural molecules are then mapped to these bottlenecks using an explicit evidence hierarchy, followed by an integrated translational appraisal and bottleneck-matched development framework. Figure 1 summarizes this organization.

## 2. TIME Features Associated with Limited PD-1/PD-L1 Response

At the immune synapse, PD-1 on activated or chronically stimulated T cells engages PD-L1 on tumor cells and antigen-presenting or myeloid cells. This interaction recruits inhibitory phosphatases, including Src homology 2 domain-containing protein tyrosine phosphatase 2 (SHP2), and attenuates T-cell receptor and CD28 signaling, cytokine production, proliferation, and cytotoxic function. Anti-PD-1 and anti-PD-L1 antibodies interrupt this inhibitory interaction, but they do not by themselves provide tumor antigen, recruit absent T cells, remove a stromal barrier, or correct severe metabolic suppression [20,21]. The TIME states below describe distinct reasons why canonical checkpoint blockade may therefore remain insufficient.

### 2.1. Failed Priming in Immune-Desert Tumors

The term immune-desert is commonly used for tumors with little evidence of productive T-cell priming or intratumoral T-cell accumulation; it should not be reduced to a single low-CD8 measurement. More precisely, the state reflects failure at an early step of the cancer-immunity cycle: Tumor antigens are not efficiently captured, processed, and cross-presented. Naive or memory T cells do not receive adequate clonal-expansion and effector-differentiation signals, and the subsequent entry of CD8+ T cells into tumor parenchyma remains limited. Conventional type 1 dendritic cells (cDC1s) are a key antigen-presenting population linking tumor antigens to cytotoxic T-cell responses. Their differentiation depends on basic leucine zipper ATF-like transcription factor 3 (BATF3) and interferon regulatory factor 8 (IRF8); they typically express X-C motif chemokine receptor 1 (XCR1) and C-type lectin domain containing 9A (CLEC9A) and have high cross-presenting capacity. cDC1s are not the only antigen-presenting route, and the location, presenting cell, and functional quality of cross-presentation must be distinguished rather than treating antigen presentation as a uniformly positive readout [20,22,23,24].

WNT/β-catenin activation is one of the better-supported mechanisms underlying immune-desert biology. In hepatocellular carcinoma models, β-catenin activation has been reported to reduce dendritic-cell recruitment and T-cell activity, and β-catenin-driven tumors resist anti-PD-1 therapy. Restoration of C-C motif chemokine ligand 5 (CCL5) can partially re-establish immune surveillance [10]. These findings explain why PD-1 blockade cannot act sufficiently when effective T-cell priming and recruitment are absent. The drug can release inhibitory signals on pre-existing T cells, but it cannot by itself create the upstream conditions required for antigen presentation, T-cell clonal expansion, and tumor infiltration. Accordingly, claims that a natural bioactive molecule improves an immune desert should be supported by effects on antigen presentation or dendritic cell (DC) and T-cell recruitment. General immune enhancement, splenic-cell changes, or increased cytokine production in vitro are insufficient [10,25].

### 2.2. Stromal and Chemokine Barriers to T-Cell Entry

The immune-excluded state differs from an immune desert. T cells may be present around the tumor, and antigen recognition may be detectable, yet these T cells fail to enter tumor nests or form sustained contact with cancer cells. The central problem is not the complete absence of immunity, but the misplacement of immunity by chemokines, spatial organization, and tissue architecture. Clinical and preclinical evidence indicates that TGF-β-associated cancer-associated fibroblast (CAF) signaling can generate an exclusionary stroma that traps CD8+ T cells in stromal regions. In the orthotopic EMT6 model, combined TGF-β and PD-L1 blockade promotes T-cell entry into tumor centers and produces stronger antitumor responses [9]. In triple-negative breast cancer, the integrin αvβ6–TGF-β–SRY-box transcription factor 4 (SOX4) pathway has also been implicated in immune escape, further supporting TGF-β as more than a fibrosis or matrix-remodeling signal; it is an important regulatory layer in immune exclusion [9,26].

CAF-associated stroma is not a single physical barrier, but a dynamic niche composed of cellular states, chemokines, and extracellular matrix. Leucine-rich repeat-containing protein 15-positive (LRRC15+) myofibroblasts can define an immunosuppressive stromal set point, suggesting that specific CAF states may themselves serve as markers and modulators of immunotherapy response [27]. In a spontaneous pancreatic cancer model, CXCL12 from fibroblast activation protein positive (FAP+) CAFs coats cancer cells and excludes T cells. After CXCR4 inhibition, T cells rapidly accumulate near cancer cells, and the combination improves the activity of PD-L1 blockade [11]. These findings indicate that the T-cell-attracting axis formed by C-X-C motif chemokine ligand 9 (CXCL9), C-X-C motif chemokine ligand 10 (CXCL10), and C-X-C motif chemokine receptor 3 (CXCR3) is important, but exclusionary chemokine axes and stromal localization cues can also determine whether T cells reach effector sites. Immune exclusion is not simply a matter of “barrier thickness”; it results from insufficient positive chemotaxis, misleading chemokine signals, CAF spatial organization, and extracellular matrix mechanics. Although the available literature includes preclinical support for dual TGF-β/vascular endothelial growth factor (VEGF) targeting with PD-1 blockade, the evidence is insufficient to develop a universal endothelial mechanism here. We treat VEGF, endothelial dysfunction, and adhesion-molecule changes as candidate layers of impaired immune entry rather than generalizing all vascular Fas ligand (FASL) or endothelial-inactivation mechanisms as universal rules [28].

### 2.3. Myeloid and Metabolic Constraints on Effector Function

Even after T cells enter tumor tissue, PD-1/PD-L1 blockade can fail when effector cells remain constrained by suppressive myeloid networks and metabolic stress. Tumor-associated macrophages (TAMs), myeloid-derived suppressor cells (MDSCs), and regulatory T cells (Tregs) can reduce T-cell cytotoxicity through nutrient competition, suppressive cytokines, arginine or tryptophan metabolism, reactive oxygen and nitrogen species (ROS/RNS), immune-checkpoint ligands, and spatial contact. Existing evidence indicates that tripartite motif-containing 28 (TRIM28) can promote anti-PD-1 resistance in non-small-cell lung cancer by enhancing MDSC recruitment, whereas apolipoprotein C1 (APOC1) inhibition can shift hepatocellular carcinoma-associated TAMs from an M2-like state toward an M1-like state and enhance anti-PD-1 efficacy. These studies show that myeloid cells are not passive background elements, but regulatory networks that determine whether PD-1 blockade has effective effector activity [29,30,31,32,33,34,35].

Metabolic restriction is another major bottleneck. Lactate accumulation, MCT4-mediated export, local acidification, and hypoxia-inducible factor 1α (HIF-1α)-associated metabolic reprogramming can constrain T-cell function while supporting suppressive myeloid or regulatory states. In prostate cancer models deficient in phosphatase and tensin homolog (PTEN) and tumor protein p53, lactate and PD-1 signaling jointly contribute to macrophage-mediated immunosuppression [31]. In colorectal cancer spheroids and MC38 models, selective MCT4 blockade reduces lactate export and, when combined with anti-PD-L1 therapy, increases immune-cell infiltration and T-cell activation [12]. Lipid metabolism may also influence ICI response: stearoyl-CoA desaturase 1 (SCD1) inhibition enhanced antitumor T-cell activity and the antitumor activity of an anti-PD-1 antibody in preclinical models [36]. In anti-PD-1-resistant microsatellite-stable colorectal cancer, vascular endothelial growth factor A has also been linked to thymocyte selection-associated high mobility group box (TOX)-dependent T-cell exhaustion [37]. Additional contributors include arginase 1 (ARG1)-mediated arginine depletion, indoleamine 2,3-dioxygenase 1 (IDO1)-associated tryptophan–kynurenine metabolism, the ectonucleoside triphosphate diphosphohydrolase 1 (CD39)/ecto-5′-nucleotidase (CD73)–adenosine axis, and cyclooxygenase-2/prostaglandin E2 (COX-2/PGE2) signaling [35,38,39,40]. IDO1 expression is cell-type and context-dependent and has been detected in tumor cells, mature dendritic cells, stromal or endothelial compartments, and macrophages in selected tumor types [41,42]. Changes in the kynurenine-to-tryptophan (Kyn/Trp) ratio should therefore not be assigned to dendritic cells, TAMs, or another specific source without cell-resolved measurements such as co-staining, cell sorting, or single-cell analysis. These pathways are relevant to resistance biology, but they should not be presented as validated natural-molecule sensitization mechanisms unless the molecule, cellular source, and combination effect are directly tested.

### 2.4. Senescence-Associated Secretory Phenotype as a Cross-State Amplifier

Therapy-induced senescence and the senescence-associated secretory phenotype (SASP) can alter the immune context of tumor tissue through interleukin-6 (IL-6), interleukin-8 (IL-8), C-C motif chemokine ligand 2 (CCL2), matrix metalloproteinases (MMPs), and other chronic inflammatory mediators. Here, SASP is better treated as a cross-state amplifying layer than as a mature TIME subtype equivalent to immune-desert, immune-excluded, or myeloid/metabolically restricted states. Studies showing that PD-L1/PD-1 blockade can improve senescence surveillance and senescence-associated phenotypes suggest a connection between checkpoint signaling and senescent-cell clearance [43]. Other work reports that tumor-cell extracellular vesicle-derived PD-L1 can promote T-cell senescence through lipid-metabolic reprogramming, indicating that senescence-like T-cell states may weaken immunotherapy responses [44]. In the senescence-associated tumor microenvironment, tumor-cell senescence-induced macrophage CD73 expression has also been reported as a metabolic immune checkpoint, suggesting potential crosstalk among SASP, myeloid cells, and adenosine signaling [45].

These signals are biologically meaningful, but the evidence boundary is narrow. SASP may amplify immune exclusion or metabolic suppression through chronic inflammation, stromal remodeling and myeloid recruitment. It does not follow that natural-product suppression of SASP enhances PD-1 therapy. The available literature does not support “natural products sensitize PD-1/PD-L1 therapy by suppressing SASP” as a main-text mechanism. Here, SASP is used to explain how TIME bottlenecks may amplify one another and to define a hypothesis layer for future causal testing [43,44,45].

### 2.5. Relationships Among TIME States

The TIME features described above represent distinct nodes of PD-1/PD-L1 treatment failure. Immune deserts arise from insufficient antigen presentation and defective T-cell priming, whereas immune-excluded tumors contain T cells that are physically barred from tumor nests by stromal elements, chemokine gradients, or tissue architecture. In myeloid-dominant or metabolically restricted tumors, effector function may be suppressed even after T cells have successfully infiltrated the lesion. The senescence-associated secretory phenotype (SASP) and senescence itself are better understood as inflammatory and tissue-remodeling forces that can exacerbate several of these barriers. Since these features frequently co-exist, resistance is rarely attributable to any single factor. Accordingly, spatial distribution, cellular composition, and functional status must be evaluated collectively and over time to capture the full picture [5,6,7,8,20,21,25,46,47].

**Figure 2:**
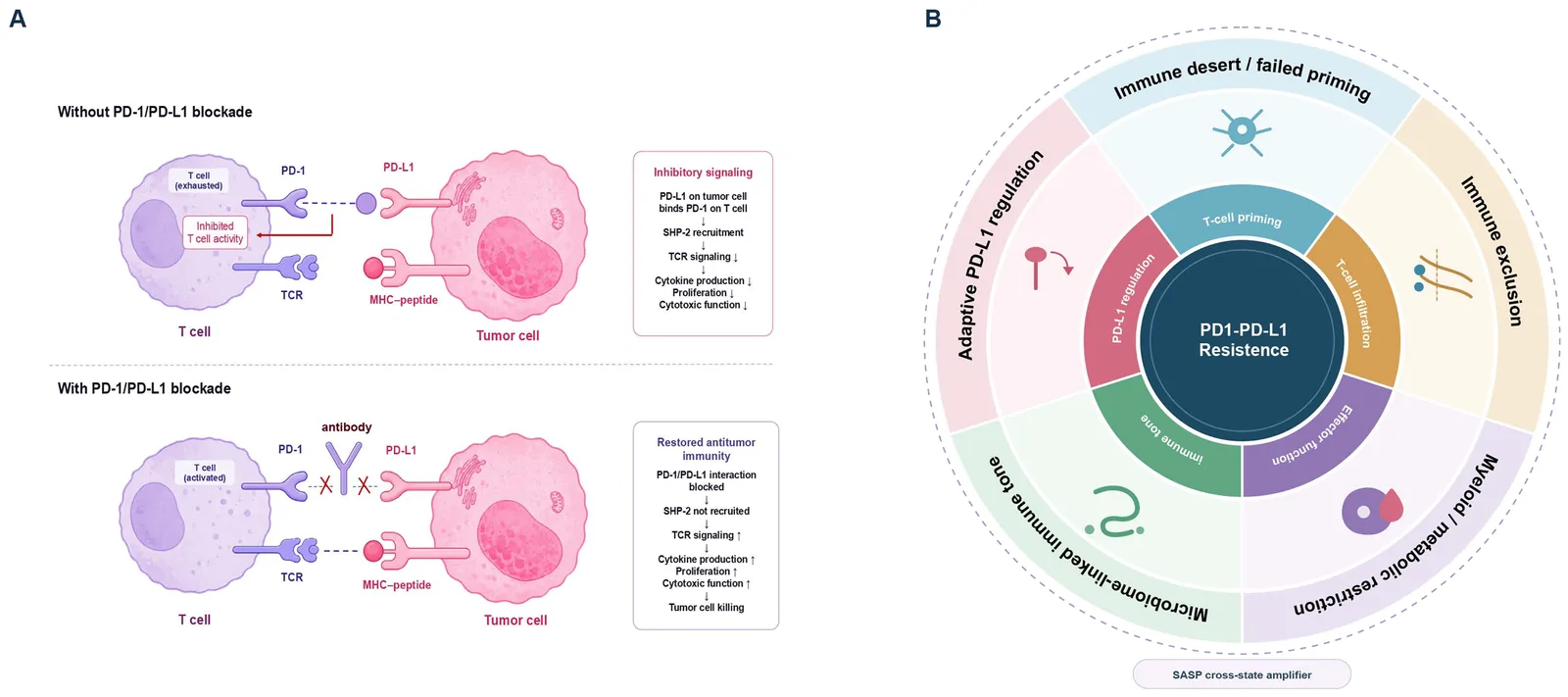
PD-1/PD-L1 checkpoint blockade and residual tumor immune microenvironmental determinants of treatment resistance. (**A**) At the tumor–T-cell interface, PD-L1 on tumor cells engages PD-1 on activated or chronically stimulated T cells. PD-1 ligation promotes inhibitory signaling, including SHP2 recruitment, and attenuates T-cell receptor and CD28 signaling, cytokine production, proliferation, and cytotoxic activity. Antibodies targeting PD-1 or PD-L1 interrupt this interaction and can relieve checkpoint-mediated inhibition. Recovery of T-cell function is conditional, however, and does not necessarily result in tumor elimination. (**B**) PD-1/PD-L1 blockade may remain ineffective when other components of the tumor immune microenvironment restrict antitumor immunity. Failed priming or an immune-desert state limits the generation of tumor-reactive T cells; immune exclusion prevents T cells from entering tumor nests; myeloid and metabolic suppression impairs effector function; microbiome-derived signals influence systemic and intratumoral immune tone; and adaptive PD-L1 regulation can maintain checkpoint pressure. These processes are not mutually exclusive and may coexist within the same tumor. The senescence-associated secretory phenotype (SASP) is shown as a cross-state amplifier because it may reinforce several of these conditions rather than constitute a separate resistance state.

These TIME features provide a biological basis for evaluating natural molecules. For each candidate, the relevant questions are whether it has been tested directly with PD-1/PD-L1 blockade, whether TIME changes were shown in an immunocompetent model, and whether target engagement or causal evidence is available. Cellular assays, omics associations, and pathway predictions are informative, but they do not establish reversal of checkpoint resistance. Figure 2 and Table 1 summarize the resistance features and the corresponding evidence boundaries.

## 3. Natural Bioactive Molecules Mapped to TIME Bottlenecks

### 3.1. Evidence Framework and Candidates with Direct Combination Evidence

Antitumor activity, immunomodulation, and checkpoint sensitization are related but not equivalent. Reduced tumor volume alone cannot distinguish checkpoint sensitization from direct cytotoxicity, metabolic stress, or nonspecific inflammation. Because PD-1/PD-L1 efficacy depends on tumor–host immune interactions, an immunocompetent model can test checkpoint-dependent CD8+ T-cell, myeloid, and microbiome effects that an immunodeficient xenograft cannot; Section 4.2 discusses model selection in detail [6,8]. Stronger studies connect a prespecified resistance state to a direct combination benefit and use depletion, blockade, rescue, target mutation, or transfer experiments to test causality. Table 2 summarizes candidates with direct PD-1/PD-L1 combination evidence or strong checkpoint-related mechanistic context.

Direct anti-PD-1/PD-L1 combinations were reported for castalagin/camu-camu, ginseng polysaccharides, ginsenoside Rh2, and curcumin–GSDME in immunocompetent mouse models [14,15,16,17]. The demethylzeylasteral study included an anti-PD-L1 arm, but its reported additional combination benefit centered on anti-CTLA-4; it therefore supports a strong USP22–PD-L1 mechanism rather than established PD-1/PD-L1 sensitization [18]. These findings are preclinical and do not constitute evidence of patient efficacy.

The reporting of exposure and active entities was uneven. The castalagin study identified castalagin as the active camu-camu fraction, confirmed its identity by liquid chromatography–mass spectrometry (LC–MS) and nuclear magnetic resonance, tested microbiome dependence, and measured serum and tumor metabolomic changes; it did not report concentrations of parent castalagin in blood or tumor [14]. The ginseng-polysaccharide study quantified plasma short-chain fatty acids, L-kynurenine, and L-tryptophan by ultra-performance liquid chromatography–mass spectrometry rather than intact-polysaccharide exposure [15]. The demethylzeylasteral study used the cellular thermal shift assay (CETSA), drug affinity responsive target stability (DARTS), and microscale thermophoresis (MST) to support USP22 binding in cell-based preparations but did not report pharmacokinetic or tissue-concentration measurements [18]. The curcumin–GSDME study specified curcumin purity, formulation, route, and dose and measured tumor pharmacodynamic effects, but not circulating or intratumoral curcumin concentrations [17]. In the Rh2 study, the dose and intratumoral immune readouts do not substitute for compound pharmacokinetics; exposure analysis remains an evidence gap [16].

### 3.2. Microbiome and Metabolic Immune Tone

The microbiome–metabolism–immune connection provides some of the clearest evidence linking natural molecules to PD-1 response. Human evidence from responder-derived FMT studies in anti-PD-1-refractory melanoma establishes the microbiome as a modifiable response axis, but not the efficacy of a specific natural molecule [13]. In immunocompetent mouse studies, castalagin/camu-camu altered microbial composition, improved the intratumoral CD8+ T-cell/FOXP3+ regulatory CD4+ T-cell ratio, and enhanced anti-PD-1 activity, including in an anti-PD-1-resistant E0771 context [14]. Ginseng polysaccharides combined with anti-PD-1 in syngeneic lung cancer models were associated with microbiome remodeling, a lower kynurenine-to-tryptophan (Kyn/Trp) ratio, increased activated peripheral CD8+ T cells, and a reduced proportion of peripheral forkhead box P3-positive (FOXP3+) Tregs [15].

These results remain preclinical. Microbial shifts can follow changes in diet, cage, tumor burden, drug exposure, or immune status, so composition alone is insufficient. Causality is strengthened when microbiome changes, metabolites, immune readouts, tumor control, and checkpoint treatment are measured in the same experiment and when antibiotics, FMT, defined colonization, or metabolite supplementation tests necessity or sufficiency. The findings should not be generalized to all polyphenols, polysaccharides, or dietary components [50,51,52,53,54].

### 3.3. PD-L1 Stability and Degradation

PD-L1 abundance is regulated through transcription, post-translational modification, ubiquitination, deubiquitination, proteasomal turnover, and autophagy–lysosomal pathways. A lower PD-L1 signal in a cell line does not by itself demonstrate restored T-cell function or greater sensitivity to checkpoint blockade. Informative studies identify the relevant target or enzyme, demonstrate altered PD-L1 stability in the appropriate cell type, include an immune-effector readout, and test a direct PD-1/PD-L1 combination [18,19,46,47].

Demethylzeylasteral has comparatively strong evidence from mouse models linking USP22 targeting, PD-L1 ubiquitin–proteasome degradation, and immune activation [18]. Its in vivo design included anti-PD-L1 and anti-CTLA-4 arms, but the reported additional combination benefit centered on anti-CTLA-4; direct PD-1/PD-L1 sensitization remains unestablished. Berberine provides in vitro and mouse evidence for a CSN5-associated PD-L1 deubiquitination mechanism and immune-infiltration changes, but no verified anti-PD-1/PD-L1 combination benefit [19]. ACT001 and baicalein remain indirect examples of signal transducer and activator of transcription 3 (STAT3)–PD-L1 regulation and autophagic PD-L1 degradation, respectively [46,47]. These candidates should not be called proven ICI sensitizers.

### 3.4. T-Cell Recruitment Through the CXCL10/CD8 Axis

T-cell recruitment lies at the intersection of failed priming, immune exclusion, and dysfunctional effector states. Tumors with weak CXCL9/CXCL10/CXCR3 signaling or dominant exclusionary signals may contain too few appropriately positioned effector cells for PD-1 blockade to reinvigorate. In the syngeneic MC38 mouse model, ginsenoside Rh2 increased intratumoral CXCL10 and CD8+ T-cell infiltration and activation in an anti-PD-L1 combination [16]. Loss of benefit after CD8 depletion and CXCR3 blockade supports a causal CXCL10/CXCR3/CD8 chain, although independent replication, broader model testing, and exposure analysis remain necessary.

Increased CD8+ abundance alone is not equivalent to restored function. Interferon-γ (IFN-γ), granzyme B, tumor necrosis factor-α (TNF-α), exhaustion state, clonality, and spatial contact with tumor cells help distinguish recruitment from effective antitumor activity. Early pharmacodynamic sampling or tumor-burden matching is needed to avoid interpreting secondary immune infiltration after tumor shrinkage as the initiating mechanism [16].

### 3.5. Myeloid and Stromal Barriers

Myeloid and stromal bottlenecks are well supported in PD-1/PD-L1 resistance biology, but direct natural-molecule intervention evidence remains limited. TGF-β/CAF signaling, LRRC15+ myofibroblasts, and the CAF–CXCL12/CXCR4 axis can restrict T-cell entry, whereas TRIM28–MDSC and APOC1–TAM studies support the contribution of myeloid recruitment and macrophage state to anti-PD-1 response [9,11,27,29,30]. A candidate proposed to repair these states should alter the suppressive function or spatial behavior of CAFs, TAMs, or MDSCs and improve a direct PD-1/PD-L1 combination, not merely change total marker abundance.

Current natural-molecule evidence in this area is mostly indirect. Berberine treatment was associated with increased CD8+ infiltration, lower intratumoral numbers of MDSCs and Tregs, and lower PD-L1 on those suppressive cells. The study did not establish a causal myeloid-reprogramming mechanism for checkpoint sensitization [19]. The ginsenoside Rg3-based liposomal carrier, evaluated chiefly as a paclitaxel-loaded formulation, improved delivery to tumor cells and MDSCs but was not tested with checkpoint blockade [55]. The targeted search did not identify a study in which a natural molecule reversed CAF–CXCL12- or TGF-β-driven spatial exclusion in combination with PD-1/PD-L1 blockade.

### 3.6. Pyroptosis as Antigen-Input Support

Immunogenic cell death (ICD) and pyroptosis can increase antigen release, danger signals, and local inflammation, but they are not equivalent to checkpoint sensitization. Antigen input must be followed by dendritic-cell uptake and cross-presentation, tumor-reactive T-cell priming, and effective recruitment; otherwise, cell death can remain immunologically unproductive or promote chronic inflammation [56].

Curcumin-induced, gasdermin E (GSDME)-dependent pyroptosis is an emerging example from a mouse model. In CT26 tumors, curcumin improved anti-PD-1 activity, and the reported effect depended on the caspase-3/GSDME axis [17]. This single study supports a specific preclinical mechanism, not a class-wide claim for curcumin or ICD-inducing natural molecules. Exposure, toxicity, reproducibility, dendritic-cell dependence, and performance in additional resistance backgrounds remain unresolved.

### 3.7. Indirect, Delivery-Related, and Hypothesis-Level Evidence

Indirect evidence remains useful for selecting mechanisms and pharmacodynamic endpoints (Table 3). Rosmarinic acid plus ginsenoside Rg1 shows pathway-associated effects without an antibody checkpoint combination [57]. A ginsenoside Rg3-based liposomal carrier and hyaluronidase-conjugated quercetin liposomes address drug distribution, MDSC targeting, or stromal penetration [55,58], but delivery does not establish target engagement or checkpoint sensitization. The enhanced permeability and retention (EPR) effect is variable and cannot by itself demonstrate that a candidate reaches a mechanism-relevant concentration in the intended tumor or immune-cell compartment.

The natural origin of a compound does not guarantee its compatibility with immunotherapy. A molecule may suppress harmful inflammation while simultaneously compromising antigen presentation or unfavorably altering myeloid and lymphoid cell populations. In the surveyed literature, direct evidence was lacking for andrographolide binding to COX-2, or for quercetin-mediated inhibition of lactate dehydrogenase A (LDHA), as mechanisms of checkpoint sensitization. Similarly, no direct evidence was found that any natural product sensitizes PD-1 therapy through suppression of the senescence-associated secretory phenotype (SASP), or that polysaccharides restore cDC1 cross-presentation. These mechanisms therefore remain unsubstantiated. Table 2 distinguishes evidence pertaining to the active entity, exposure, and target engagement from immune pharmacodynamic readouts.

### 3.8. Evidence Summary and Next Validation

**Figure 3:**
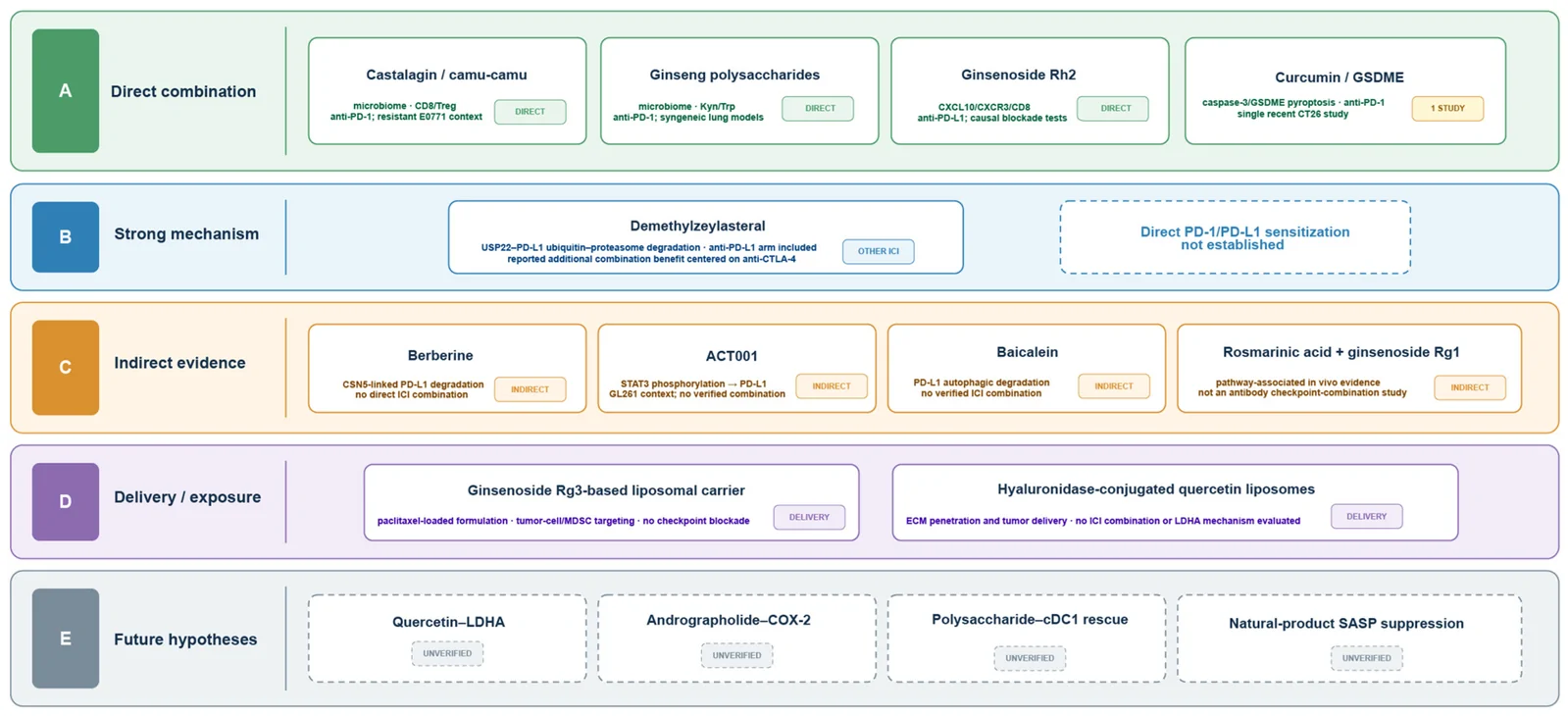
Strength and scope of evidence linking natural bioactive molecules to PD-1/PD-L1 therapy. Candidates are grouped according to the highest level of evidence supported by the reported experiments. (**A**) Castalagin/camu-camu, ginseng polysaccharides, ginsenoside Rh2, and the curcumin–GSDME axis have been tested directly with anti-PD-1 or anti-PD-L1 antibodies in immunocompetent mouse models. The curcumin–GSDME evidence is derived from a single recent study and therefore requires independent confirmation. (**B**) Demethylzeylasteral has comparatively strong evidence for USP22-associated PD-L1 ubiquitination and proteasomal degradation. Although the study included an anti-PD-L1 treatment arm, the reported additional combination benefit centered on anti-CTLA-4; direct sensitization to PD-1/PD-L1 blockade has therefore not been established. (**C**) Berberine, ACT001, baicalein, and rosmarinic acid combined with ginsenoside Rg1 are supported by indirect evidence of PD-L1 regulation, immune modulation, or checkpoint-associated signaling, without a verified anti-PD-1/PD-L1 antibody combination. (**D**) The ginsenoside Rg3-based liposomal carrier and hyaluronidase-conjugated quercetin liposomes address tumor delivery, stromal penetration, or cellular targeting but do not demonstrate checkpoint sensitization. (**E**) Quercetin–LDHA, andrographolide–COX-2, polysaccharide-mediated cDC1 rescue, and natural-product suppression of the senescence-associated secretory phenotype remain unverified hypotheses in the context of PD-1/PD-L1 therapy. These categories describe the strength of evidence for the indicated claim and should not be interpreted as a quantitative ranking of therapeutic efficacy.

The available evidence differs substantially in experimental depth and in its relevance to PD-1/PD-L1 therapy. Figure 3 groups the candidates according to the strongest claim supported by the reported experiments. Direct antibody-combination evidence is distinguished from checkpoint-related molecular mechanisms, indirect immune or PD-L1 regulation, delivery studies, and hypotheses that have not been tested in an appropriate combination model. Placement in a higher category indicates stronger experimental support for the specific claim shown; it does not imply clinical efficacy or a class-wide effect.

## 4. Translational Appraisal and Bottleneck-Matched Development

### 4.1. From Hypothesis Generation to Clinical Relevance

Translational prioritization depends not only on the reported biological activity of a candidate, but also on the strength of evidence supporting its proposed mechanism and its relevance to PD-1/PD-L1 resistance. Accordingly, we classify the available evidence into five hierarchical levels, ranging from hypothesis-generating observations to clinically supported interventions. This framework provides a practical basis for interpreting current findings and identifying the experiments required for further translational development (Table 4).

For evidence categorized as Level 1 or 2, the logical next step is direct biochemical or cellular validation, followed by in vivo testing in immunocompetent models. Level 3 requires the inclusion of a direct anti-PD-1/PD-L1 combination, immune readouts, and formal causal assessment. At Level 4, replication across multiple resistance-matched models is essential, along with measurements of drug exposure, target engagement, and predefined biomarkers. Level 5 applies only when the native molecule itself has progressed into human clinical studies. Of note, clinical microbiome investigations do not, on their own, constitute clinical evidence for any individual natural molecule [13,14,15,16,17,18,19,46,47,55,57,58].

### 4.2. Immunocompetent Models and Spatial Context

Because PD-1/PD-L1 blockade depends on the host immune system, model immune competence is the first translational gate. Immunodeficient xenografts can be useful for preliminary assessment of tumor inhibition or drug exposure, but they cannot directly evaluate the roles of CD8+ T cells, dendritic cells (DCs), TAMs, MDSCs, Tregs, or the PD-1/PD-L1 axis in combination efficacy. Subcutaneous syngeneic models are efficient and reproducible and are useful for early combination screening. However, they may underestimate organ-specific vascular architecture, stromal structure, metabolic gradients, bile acids, or gut microbiome effects, and they often do not fully reproduce the spatial ecology of liver, lung, pancreatic, or colorectal tumors. Orthotopic, spontaneous, genetically engineered, humanized, and patient-derived platforms each have strengths and limitations; none should be treated as a single “best” model [6,8,59,60,61].

Model design should match the TIME bottleneck being claimed. Microbiome studies require control of diet, cage effects, and antibiotic exposure, together with fecal microbiota transplantation or defined microbial colonization experiments when causality is claimed [13,14,15]. Metabolic studies should connect intratumoral metabolites and transporters to immune function, as in work linking MCT4 blockade, lactate export, anti-PD-L1 treatment, and T-cell activation [12]. Myeloid or stromal claims require spatial evidence rather than total marker abundance. Multiplex immunofluorescence and digital pathology define tissue organization; flow cytometry and single-cell sequencing resolve cell states; spatial transcriptomics evaluates neighborhoods and localization. Patient-derived micro-organospheres and tumor-immune organoids can supplement model stratification when immune, stromal, and exposure conditions are preserved or reconstructed [59,60,61], but they do not reproduce systemic pharmacokinetics, tissue distribution, or gut–liver biotransformation.

The model should also contain the resistance state being studied. If a model is highly sensitive to anti-PD-1 at baseline, improved efficacy from a candidate may reflect general antitumor additivity rather than resistance overcoming. If a model lacks immune priming entirely, increasing dose intensity may not reveal the intended mechanism. A more informative design includes sensitive, low-response, or resistant backgrounds and explains differences using baseline TIME features. For natural molecules, it is especially important to avoid extrapolating from a combination benefit in an easy-to-treat subcutaneous model to immune-excluded, myeloid-dominant, or metabolically restricted tumors.

### 4.3. Exposure, Target Engagement and Dose Window

A common translational failure point for natural molecules is the absence of verifiable in vivo exposure and target engagement, even when in vitro activity is clear. Systemic dosing is not equivalent to tumor exposure, and tumor exposure is not equivalent to target engagement in the relevant cell type. Claims of direct action on USP22, CSN5, PD-L1, STAT3, or another node require orthogonal target-engagement evidence where feasible. CETSA, DARTS, pull-down, competition, microscale thermophoresis, and mutation or rescue experiments address different parts of this question [18,19,46,47,62,63]. A mechanistic chain that links target perturbation, PD-L1 turnover, and immune response is stronger than an isolated decrease in PD-L1, but it does not replace direct checkpoint-combination evidence.

The dose window is equally important. Low doses may mainly affect the microbiome or immune modulation, whereas high doses may produce cytotoxicity, broad anti-inflammatory effects, or nonspecific stress; these scenarios have different meanings for TIME biology. A molecule that reduces PD-L1 or induces pyroptosis in vitro may not reach the same concentration safely in vivo. Delivery systems can improve exposure, but they cannot replace mechanistic proof. Lactate oxidase nanocapsules and the ginsenoside Rg3-based liposomal carrier suggest that delivery strategies can alter metabolic or myeloid-related exposure [55,64]. Quercetin liposomes plus hyaluronidase are more appropriately used as supplementary evidence for stromal penetration and delivery problems, not as evidence for an LDHA-ICI mechanism or PD-1 sensitization [58]. Nanodelivery, biomimetic delivery, and liposomes address only part of the exposure problem; researchers must still show where the candidate arrives, which cells it acts on, and whether it changes the prespecified TIME bottleneck [55].

For the appraisal used here, the administered parent material is distinguished from circulating metabolites, microbial transformation products, and delivery-associated species [14,15,55,58,64]. The molecular entity that contacts tumor cells, immune cells, or the gut microbiome may differ from the structure tested in vitro. Pharmacokinetic, tissue-distribution, and pharmacodynamic sampling should therefore be aligned on the same timeline. This pharmacokinetic/pharmacodynamic (PK/PD) design also needs to allow for delayed immune effects, including cytokine changes, immune-cell recruitment, and functional T-cell activation, rather than assuming that compound exposure and immune response peak together.

### 4.4. Material Quality and Reproducibility

Materials grouped as natural bioactive molecules are not analytically equivalent. Extracts, formulas, crude polysaccharides, purified monomers, derivatives, metabolites, and delivery formulations differ in composition, pharmacokinetics, immune effects, and reproducibility. Mixing these materials together can create mechanistic misattribution. Polysaccharide immune effects may depend on molecular weight, monosaccharide composition, branching, solubility, endotoxin contamination, host microbiome, and route of administration. Polyphenol effects may be mediated largely through microbial metabolites or microbe binding. Purified small molecules require stronger target-engagement and structure–activity support. Material standardization is part of translational evidence, not a technical afterthought [14,15,65].

For PD-1/PD-L1 combination studies, quality control should at minimum report source, batch, extraction and purification procedures, purity, structural characterization, residual solvent or endotoxin testing, storage conditions, route, dose, frequency, antibody timing, and combination window. For formulas or complex extracts, quality markers and ultra-high-performance liquid chromatography (UHPLC) or liquid chromatography–mass spectrometry (LC–MS) fingerprints can support compositional consistency; chemical quality control does not replace immune-mechanism validation. For polysaccharide candidates, microbiome, Kyn/Trp, and immune readouts do not compensate for unresolved batch composition, structural parameters, or microbiome dependence [15]. Without such information, later studies cannot determine whether failure reflects candidate inefficacy, inadequate exposure, material inconsistency, or mismatch between model and resistance bottleneck.

Polysaccharides and extracts require particular attention. Endotoxin contamination can produce false immune activation, trace bioactive impurities can be mistaken for the effect of the main component, and batch-to-batch variation in monosaccharide ratios or molecular-weight distribution can alter microbiome responses. Residual lipopolysaccharide (LPS) can confound macrophage-polarization, dendritic-cell-maturation, and inflammatory readouts attributed to a polysaccharide preparation. Endotoxin removal, quantitative testing, and appropriate controls are therefore needed to distinguish intrinsic immunomodulatory activity from contamination-driven effects [65,66]. Reporting these controls in the main study makes immune readouts and cross-laboratory replication easier to interpret.

### 4.5. Timing, Controls and Pharmacodynamic Endpoints

Natural molecules should not be combined with PD-1/PD-L1 inhibitors in designs that simply administer treatment and measure tumor volume. Different bottlenecks imply different timing windows. Microbiome or stromal remodeling may require pretreatment; PD-L1 degradation may be suited to concurrent or short-term combination; ICD/pyroptosis may need timing that aligns antigen presentation and T-cell recruitment; and myeloid or Treg modulation may require sustained pharmacodynamic monitoring. If all candidates are tested with the same dosing sequence, negative findings may reflect design mismatch rather than absence of mechanism.

Pharmacodynamic endpoints must also match the bottleneck. Microbiome–metabolism studies should jointly monitor microbial composition, key microbes or metabolites, the Kyn/Trp ratio, CD8^+^T-cell/Treg balance, and antibiotic or FMT rescue [13,14,15]. PD-L1-stability studies should measure PD-L1 protein half-life, ubiquitination or autophagic degradation, target engagement, and antibody combination response [18,19,47]. T-cell-recruitment studies should report CXCL9/CXCL10, CXCR3, CD8+ T-cell spatial position, effector/exhaustion state, and causal tests such as CD8 depletion or chemokine-axis blockade; the Rh2 study already includes these key causal elements but still requires independent replication and exposure validation [16]. Myeloid/stromal studies require spatial states of TAMs, MDSCs, and CAFs, not only M1/M2 markers [9,11,29,30]. ICD/pyroptosis studies should include calreticulin (CRT), high-mobility group box 1 (HMGB1), adenosine triphosphate (ATP), GSDME/caspase-3 dependence, and CD8 dependence; the curcumin–GSDME study provides an emerging signal but needs further model and exposure validation [17].

Combination designs require negative and mechanistic controls. Minimum groups include anti-PD-1/PD-L1 monotherapy, natural-molecule monotherapy, combination treatment, and toxicity- or dose-matched controls. If the candidate has direct cytotoxicity, tumor-burden matching or early pharmacodynamic sampling should be added to avoid interpreting secondary immune infiltration after tumor shrinkage as the primary mechanism. Microbiome studies need cage randomization and fecal-transfer controls [13,14,15]. Target-degradation studies benefit from protein rescue or target-mutant rescue [18,19,47]. Only when these designs are present does a combination strategy approach a testable translational plan.

### 4.6. Integrated Development and Decision Workflow

Table 5 translates the appraisal criteria into a stepwise decision framework. The process begins by defining the dominant resistance state and then selecting a candidate whose available evidence maps to that state. Before any direct checkpoint combination can be interpreted, the identity and purity of the material must be confirmed, along with adequate exposure in the relevant compartments. Causal perturbation experiments and pharmacodynamic biomarkers tailored to the bottleneck are required to distinguish a primary immune mechanism from secondary changes driven by tumor shrinkage. A candidate may advance only when the preparation is reproducible, exposure is sufficient, a prespecified combination contrast shows improvement over both monotherapies in a poorly responsive or resistant model, and the causal and pharmacodynamic evidence converge. If these conditions are not met, the claims should be narrowed, the study redesigned, or the program discontinued.

## 5. Conclusions

Natural bioactive molecules may provide mechanism probes and investigational adjuncts for PD-1/PD-L1-resistant solid tumors, but current evidence is dominated by preclinical studies. Direct anti-PD-1/PD-L1 combination evidence in immunocompetent mice should be distinguished from mechanistically strong studies using another checkpoint, from single-agent PD-L1 regulation, and from delivery-only findings [14,15,16,17,18,19,46,47,55,57,58]. This distinction is more informative than ranking candidates by chemical class.

The central interpretive requirement is to separate a well-supported resistance mechanism from evidence that a specific natural material modifies that mechanism. PD-L1 downregulation, increased CD8+ abundance, microbiome shifts, or improved tumor delivery are individually insufficient. Confidence increases when the tested preparation is defined, the relevant tissue or cell population is exposed, the proposed target is engaged, the TIME bottleneck changes before or independently of tumor shrinkage, and depletion, blockade, rescue, or transfer experiments support causality [14,15,16,17,18,19,46,47,55,57,58].

The current data support investigational adjunctive strategies, not replacement of approved PD-1/PD-L1 antibodies. Human FMT studies provide proof of concept that the microbiome can modify checkpoint response in a subset of patients, but they do not establish clinical efficacy for the natural molecules reviewed here or prove a survival benefit from the cited single-arm study [13]. Clinical translation will also require indication-specific biomarker logic, because PD-L1 assay, score, cutoff, and treatment context vary across tumors, and PD-1 expression is not a general tumor-cell eligibility threshold [1].

Future studies should focus on a select few candidates with well-verified exposure profiles and appropriate disease-state matching, rather than expanding compound libraries. Meaningful progress will require reproducible material quality, a model that genuinely embodies the claimed resistance state, predefined endpoints demonstrating superiority over each monotherapy, and converging causal and pharmacodynamic evidence. Moreover, a formal interaction analysis is essential before any effect can be attributed to pharmacological synergy. If the combination fails to achieve relevant exposure, target engagement, or mechanism-based benefit, the claims should be qualified, the study redesigned, or the program terminated. These criteria are designed to bridge mechanistic insights with testable preclinical development while ensuring that clinical assertions remain grounded in the available evidence.

## Figures and Tables

**Table 1:** Resistance-associated TIME states and evidence boundaries for natural-molecule mechanism matching.

TIME State	Dominant Bottleneck	Representative Biomarkers/Readouts	Relevance to PD-1/PD-L1 Resistance	Natural Molecule Intervention Evidence Status	Caution	Supporting Citation(s)
Immune desert/priming failure	cDC1 recruitment and cross-presentation failure	cDC1, BATF3, IRF8, XCR1, CLEC9A, beta-catenin activation, CCL5/CCL4-related DC recruitment, low CD8 priming	PD-1 blockade has limited substrate when antigen presentation and T-cell priming are absent or weak	Strong TIME biology; no verified natural-molecule cDC1 rescue identified in the current evidence set	Do not claim polysaccharides or natural products rescue cDC1 cross-presentation without direct in vivo evidence	[10,22]
Immune exclusion/stromal barrier	T cells remain outside tumor nests because of CAF, TGF-beta, CXCL12/CXCR4, collagen, and stromal positioning	TGF-beta fibroblast signature, CAF/LRRC15 state, CXCL12, CXCR4, collagen, spatial CD8 localization	Spatial exclusion can prevent reinvigorated T cells from contacting tumor cells despite checkpoint blockade	Strong TIME biology; natural-molecule repair of CAF/TGF-beta/CXCL12 exclusion remains indirect or future-facing	Do not state that natural products directly repair stromal exclusion unless direct combination and spatial evidence are available	[9,11,26,27,28]
Myeloid-dominant suppression	TAM, MDSC, and Treg networks biochemically and spatially suppress effector T cells	TAM, MDSC, Treg, ARG1, IDO1, CD39/CD73, adenosine, M2/M1 markers, TRIM28-MDSC, APOC1-TAM	Suppressive myeloid states can limit PD-1/PD-L1 efficacy even when T cells are present	Strong TIME biology; natural-molecule myeloid intervention evidence is mostly indirect or delivery-related	Do not convert immune-cell marker changes into proven ICI sensitization	[29,30,31,32,33,34,35,38,39,40,41,42]
Metabolic restriction	Lactate export, acidosis, and nutrient competition impair T-cell and myeloid function	MCT4, lactate, low pH, HIF-1alpha, Kyn/Trp, SCD1/lipid stress, T-cell activation markers	Metabolic restriction can keep T cells functionally disabled despite checkpoint blockade	Strong TIME biology; ginseng polysaccharides support a microbiome-Kyn/Trp axis; quercetin-LDHA claim remains unsupported	Do not claim quercetin-LDHA or broad natural-product metabolic reprogramming as ICI resistance reversal	[12,15,31,36]
Microbiome-linked immune tone	Gut microbiome and metabolites alter systemic immune permissiveness	Microbiome composition, responder taxa, FMT response, Kyn/Trp ratio, CD8/Treg balance	Clinical FMT studies and preclinical natural-molecule studies support microbiome as a modifiable PD-1 response axis	Best-supported natural-molecule module for castalagin/camu-camu and ginseng polysaccharides in preclinical models	Do not infer clinical efficacy of natural products from microbiome association	[13,14,15]
PD-L1 adaptive regulation	PD-L1 expression, stability, ubiquitination, deubiquitination, or degradation modulates immune escape	PD-L1 protein half-life, USP22, CSN5, STAT3, ubiquitination, autophagic degradation	PD-L1 regulatory mechanisms can influence tumor-cell immune evasion and response to PD-1/PD-L1 blockade	Demethylzeylasteral has strong USP22–PD-L1 degradation evidence with checkpoint-related immunotherapy-combination context; direct anti-PD-L1-combination evidence remains unconfirmed	Do not treat any PD-L1 decrease as proven ICI sensitization	[18,19,48,49]
SASP/therapy-induced senescence overlay	Chronic inflammatory and tissue-remodeling signals may amplify exclusion or myeloid recruitment	IL-6, IL-8, CCL2, MMPs, CD73, senescence-associated PD-L1 or T-cell senescence markers	SASP may modulate TIME but is not a validated natural-product-PD-1 sensitization mechanism	Future-facing overlay only for natural molecules; not a validated natural-product-PD-1 mechanism	Do not claim natural products suppress SASP to sensitize PD-1 therapy	[43,44,45]

**Table 2:** Natural molecules with direct PD-1/PD-L1 combination evidence or strong checkpoint-related mechanistic context.

Molecule/ Material	Source/Type and Active-Entity or Exposure Evidence	Cancer Model	ICI Used	Main Mechanism	Immune Readouts	Evidence Level	Caution	Supporting Citation(s)
Castalagin/camu-camu	Polyphenol isolated from camu-camu; identity confirmed by liquid chromatography–mass spectrometry and nuclear magnetic resonance; serum/tumor metabolomics reported, but parent blood/tumor concentration was not reported	Syngeneic mouse tumor models including anti-PD-1-resistant E0771 context; FMT-linked experiments	anti-PD-1	Microbiome-linked immune tone and CD8/Treg balance	Microbiota shifts, intratumoral CD8+/FOXP3+CD4+ balance, antitumor immune readouts	Direct preclinical PD-1 combination evidence	Preclinical; not patient efficacy; microbiome causality should not be generalized to all polyphenols	[14]
Ginseng polysaccharides	Ginseng polysaccharide preparation; plasma short-chain fatty acids and the kynurenine/tryptophan (Kyn/Trp) ratio were measured, not intact polysaccharide exposure	Syngeneic lung cancer models; FMT and patient microbiome association components	anti-PD-1	Microbiome remodeling and Kyn/Trp-linked metabolic immune modulation	Activated CD8+ T cells, reduced Foxp3+ Tregs, Kyn/Trp decrease, microbiome readouts	Direct preclinical PD-1 combination evidence	Preclinical; material standardization and polysaccharide structure/batch details remain important	[15]
Demethylzeylasteral	Tripterygium-derived small molecule; cell-based USP22 engagement supported by cellular thermal shift assay, drug affinity responsive target stability, and microscale thermophoresis; no pharmacokinetic or tissue-concentration data	MC38 tumor model	anti-PD-L1 and anti-CTLA-4 arms were included; reported additional combination benefit centered on anti-CTLA-4	USP22-associated PD-L1 ubiquitin-proteasome degradation	Antitumor immunity readouts including T-cell and suppressive-cell changes as reported	Strong PD-L1 degradation and in vivo immune mechanism with anti-CTLA-4 combination context; not direct PD-1/PD-L1-combination evidence	Direct PD-1/PD-L1-combination efficacy remains unverified; clinical efficacy is unproven.	[18]
Curcumin/GSDME	>98% curcumin in a 1,2-distearoyl-sn-glycero-3-phosphoethanolamine–methoxy poly(ethylene glycol) 2000 (DSPE-mPEG2000) formulation; route and dose reported; circulating and intratumoral curcumin concentrations were not measured	CT26 colorectal cancer model in a recent single preclinical study	anti-PD-1	Caspase-3/GSDME-dependent pyroptosis as antigen-input support	GSDME dependency and immune-combination readouts reported	Emerging single-study preclinical ICI-combination evidence	Single new study; target engagement and broader model reproducibility not established	[17]
Ginsenoside Rh2/CXCL10	Ginseng-derived saponin; dose and tumor immune pharmacodynamics reported; compound pharmacokinetics remain unverified	MC38 syngeneic tumor model	anti-PD-L1	TANK-binding kinase 1 (TBK1)–interferon regulatory factor 3 (IRF3)–CXCL10-linked CD8+ T-cell recruitment	CD8+ infiltration, proliferation, and activation; CD8-depletion and CXCR3-blockade validation	Direct preclinical anti-PD-L1-combination evidence with causal immune validation	Preclinical; independent replication, exposure/target-engagement analysis, and broader model validation remain necessary.	[16]

**Table 3:** Indirect, delivery-related, and hypothesis-level evidence for natural-molecule TIME mechanisms.

Molecule/ Formulation	Proposed Mechanism	Model/ Evidence Type	Direct ICI Combination Verified? Yes/No	Evidence Level	Overclaim Risk	Recommended Use	Supporting Citation(s)
Berberine	CSN5-linked PD-L1 deubiquitination blockade and PD-L1 degradation with immune infiltration changes	Cell, target-binding, and Lewis lung tumor model without verified anti-PD-1/PD-L1 combination	No	Indirect in vivo TIME/PD-L1 mechanism	Could be overstated as proven ICI sensitizer or macrophage-reprogramming mechanism	Use as cautious main-text PD-L1 degradation mechanism; do not claim direct ICI efficacy	[19]
ACT001	STAT3 phosphorylation inhibition leading to reduced PD-L1 expression	Glioblastoma cell and GL261 in vivo model; direct anti-PD-1 combination not verified	No	Indirect PD-L1 regulatory mechanism	Could be overstated as anti-PD-1 synergy	Use as indirect STAT3-PD-L1 example	[46]
Baicalein	CD274/PD-L1 targeting and autophagic degradation	Target/degradation evidence; direct ICI combination not verified in available evidence	No	Indirect mechanism evidence; Cell/target mechanism; supplementary evidence	Could be overstated as validated ICI sensitization	Use as supplementary PD-L1 degradation evidence only	[47]
Rosmarinic acid + ginsenoside Rg1	COX-2 and PD-1/PD-L1-associated pathway modulation in metastasis context	MC38 colon cancer lung metastasis model; not antibody checkpoint-combination study	No	Indirect pathway-associated in vivo evidence	Could be confused with direct anti-PD-1/PD-L1 antibody combination	Use as supplementary pathway evidence; no ICI-combination claim	[57]
Rg3-lipo	Biomimetic delivery to tumors and MDSCs	Preclinical nanodelivery study	No	Delivery/exposure evidence	Could be overstated as natural-molecule ICI sensitization or target engagement	Use only for delivery/exposure barrier discussion	[55]
Quercetin liposomes	Liposome plus hyaluronidase delivery/stromal penetration in pancreatic cancer context	Delivery evidence; full-text and immunotherapy-relevant LDHA mechanism not verified	No	Delivery/future validation evidence	High risk if linked to quercetin-LDHA ICI reversal	Use only as delivery example; exclude LDHA-ICI claim	[58]
Natural-product SASP suppression	Hypothesized reduction of SASP-mediated immunosuppression	No verified natural-product plus PD-1 sensitization mechanism	No	Hypothesis-level or unsupported mechanism	Would overstate unverified SASP-PD-1 sensitization	Mention only as future-facing hypothesis or omit	No positive supporting evidence identified.
Quercetin-LDHA	Hypothesized LDHA/lactate immunotherapy mechanism	No verified immunotherapy-relevant claim in current evidence map	No	Hypothesis-level or unsupported mechanism	Would falsely imply metabolic ICI sensitization	Exclude from supported mechanism claims	No positive supporting evidence identified.
Andrographolide-COX2	Hypothesized direct COX-2 binding/modification in ICI context	No verified direct support in current evidence map	No	Hypothesis-level or unsupported mechanism	Would falsely imply COX-2/PGE2 ICI sensitization	Exclude	No positive supporting evidence identified.
Polysaccharide-cDC1 rescue	Hypothesized rescue of cDC1 cross-presentation	No verified in vivo natural-polysaccharide cDC1 rescue in ICI context	No	Hypothesis-level or unsupported mechanism	Would overstate immune desert repair	Exclude from supported mechanism claims	No positive supporting evidence identified.

**Table 4:** Evidence hierarchy for prioritizing natural bioactive molecules in PD-1/PD-L1 resistance.

Evidence Level	Evidence Type	What It Can Support	What It Cannot Support	Representative Examples from This Review	Required Next Validation	Supporting Citation(s)
Level 1	Docking, network pharmacology, correlation-only, or public database enrichment	Hypothesis generation and candidate pathway selection	Mechanism proof, target engagement, TIME remodeling, or ICI sensitization	Unsupported/future-only mechanisms such as quercetin-LDHA or andrographolide-COX2 in this review	Direct biochemical, cellular, and model-based validation before evidence-level upgrading	No positive supporting evidence identified.
Level 2	Cell-based mechanism, target degradation, or pathway modulation without adequate in vivo ICI context	A plausible molecular mechanism or pharmacodynamic marker	In vivo TIME repair, direct PD-1/PD-L1 sensitization, or clinical relevance	ACT001-STAT3-PD-L1; baicalein-PD-L1 autophagic degradation	Immune-competent in vivo testing, target engagement, and direct ICI combination	[46,47]
Level 3	In vivo tumor or delivery model without verified anti-PD-1/PD-L1 antibody combination	In vivo biological activity, exposure strategy, or indirect TIME modulation	ICI-combination efficacy or resistance reversal	Demethylzeylasteral (anti-CTLA-4 combination only); berberine; rosmarinic acid + ginsenoside Rg1; Rg3-lipo; quercetin liposomes	Add anti-PD-1/PD-L1 groups, immune readouts, exposure and causality testing	[18,19,55,57,58]
Level 4	Immune-competent or mechanism-appropriate direct PD-1/PD-L1 combination with immune readouts	Preclinical prioritization as a candidate ICI-combination strategy	Clinical efficacy or broad class effect cannot be inferred from Level 4 evidence.	Castalagin/camu-camu; ginseng polysaccharides; ginsenoside Rh2; curcumin/GSDME (single-study emerging evidence)	Replicate across models, verify exposure/target engagement, and define biomarkers	[14,15,16,17]
Level 5	Clinical trial, patient cohort, or clinical intervention with mechanistic support	Clinical or translational relevance of a pathway or intervention	Natural-product efficacy unless the natural product itself was tested clinically with ICI	FMT in anti-PD-1-refractory melanoma as microbiome-axis benchmark	Natural-molecule-specific clinical safety, pharmacodynamic, and efficacy studies	[13]

**Table 5:** Decision framework for advancing natural-molecule combinations with PD-1/PD-L1 blockade.

Development Stage	Gate	Minimum Evidence/Readouts	Decision Rule
1. Define resistance state	Which TIME bottleneck limits response?	Baseline spatial and immune profiling; metabolism or microbiome assays when relevant [9,10,11,12,13,22,29,30,31].	Downgrade until the model or patient subset contains the claimed bottleneck.
2. Select candidate	Does evidence map to that bottleneck?	Claim-level review of direct mechanism, model, checkpoint partner, immune readouts, and evidence level [14,15,16,17,18,19,46,47,55,57,58].	Keep popularity- or class-based choices as hypothesis-only.
3. Verify material and exposure	Is the tested entity reproducible and bioavailable?	Source, batch, purity/fingerprint, endotoxin control, pharmacokinetics, relevant-compartment concentration, and target engagement [15,18,19,46,55,58,62,63,64,65,66].	Do not claim mechanism if the active entity, exposure, or target engagement is unresolved.
4. Test direct combination	Does the candidate improve checkpoint response beyond both monotherapies in a poorly responsive or resistant model?	Natural molecule, checkpoint inhibitor, and combination groups in an immunocompetent or mechanism-matched model; prespecified combination contrast; immune and tumor readouts [14,15,16,17].	Downgrade cell-only, xenograft-only, or tumor-volume-only evidence; do not claim synergy without formal interaction analysis.
5. Validate causality	Is the proposed bottleneck necessary?	Depletion, blockade, rescue, transfer/FMT, target mutation, degradation, or spatial validation matched to the mechanism [13,14,15,16,17,18,19,47].	Distinguish primary mechanism from toxicity or secondary changes after tumor shrinkage.
6. Integrate biomarkers and decide	Is the effect exposure-linked, reproducible, and monitorable?	Baseline, early on-treatment, and endpoint biomarkers integrated with material quality, exposure, efficacy, and safety.	Advance only when evidence converges; otherwise narrow the claim, redesign, or discontinue.

## Data Availability

No new data were created or analyzed in this study. Data sharing is not applicable to this article.
